# Spatial Resolution of *Mycobacterium tuberculosis* Bacteria and Their Surrounding Immune Environments Based on Selected Key Transcripts in Mouse Lungs

**DOI:** 10.3389/fimmu.2022.876321

**Published:** 2022-05-18

**Authors:** Anastasia Magoulopoulou, Xiaoyan Qian, Todia Pediatama Setiabudiawan, Sergio Marco Salas, Chika Yokota, Martin E. Rottenberg, Mats Nilsson, Berit Carow

**Affiliations:** ^1^ Science for Life Laboratory, Department of Biochemistry and Biophysics, Stockholm University, Solna, Sweden; ^2^ Department of Microbiology, Tumor and Cell Biology and Centre for Tuberculosis Research, Karolinska Institutet, Solna, Sweden

**Keywords:** *Mycobacterium tuberculosis*, pathogen–host interaction (PHI), granuloma, *in situ* sequencing, automated bacteria identification, distance-based transcript analysis, automated tuberculous lesion identification, innate immune activation

## Abstract

*Mycobacterium tuberculosis* (Mtb) bacilli are the causative agent of tuberculosis (TB), a major killer of mankind. Although it is widely accepted that local interactions between Mtb and the immune system in the tuberculous granuloma determine whether the outcome of infection is controlled or disseminated, these have been poorly studied due to methodological constraints. We have recently used a spatial transcriptomic technique, *in situ* sequencing (ISS), to define the spatial distribution of immune transcripts in TB mouse lungs. To further contribute to the understanding of the immune microenvironments of Mtb and their local diversity, we here present two complementary automated bacteria-guided analysis pipelines. These position 33 ISS-identified immune transcripts in relation to single bacteria and bacteria clusters. The analysis was applied on new ISS data from lung sections of Mtb-infected C57BL/6 and C3HeB/FeJ mice. In lungs from C57BL/6 mice early and late post infection, transcripts that define inflammatory macrophages were enriched at subcellular distances to bacteria, indicating the activation of infected macrophages. In contrast, expression patterns associated to antigen presentation were enriched in non-infected cells at 12 weeks post infection. T-cell transcripts were evenly distributed in the tissue. In Mtb-infected C3HeB/FeJ mice, transcripts characterizing activated macrophages localized in apposition to small bacteria clusters, but not in organized granulomas. Despite differences in the susceptibility to Mtb, the transcript patterns found around small bacteria clusters of C3HeB/FeJ and C57BL/6 mice were similar. Altogether, the presented tools allow us to characterize in depth the immune cell populations and their activation that interact with Mtb in the infected lung.

## Introduction

Tuberculosis (TB) still causes 1.5 million deaths every year. Infection with *Mycobacterium tuberculosis* (Mtb), the causative agent of TB, occurs when bacilli are inhaled and phagocytized by lung alveolar macrophages that initiate the formation of a granuloma, the pathological hallmark of TB (1, 2). Host–bacteria interactions in the granuloma are required for bacteria containment, persistence, or clearance (3). However, changes in the granuloma microenvironment could also enable Mtb growth and systemic dissemination and transmission (4). If the integrity of the granuloma is lost due to impaired immunity, reactivation of Mtb leads to profound damaging of the lung structure and the transmission of Mtb (3, 5). A major impediment to the development of new TB vaccines and therapies is the incomplete understanding of the local protective immunity in the lungs, which is the primary organ targeted by infection (6–8).

Granulomas show an important histological and immune heterogeneity. While the histological features of granulomas have been well characterized, the immune mechanisms that underlie variable granuloma dynamics and clinical outcomes of TB infection remain to be further elucidated (9, 10). Importantly, granulomas in the same tissue may evolve independently of each other and studies in non-human primates demonstrated that during active TB, sterile lesions could be found in the same lung (11). Granulomas have also been shown to provide an immunosuppressive milieu restricting anti-Mtb immune effector functions (12). We have previously described the accumulation of immune suppressive transcripts in the center of necrotic granulomas of Mtb-infected C3HeB/FeJ mice. Moreover, another study demonstrated that granuloma-localized TGF-β signaling impaired the effector function of CD4 T cells in the lung tissue of Mtb-infected C57BL/6 mice (13, 14). T cells in the lymphocyte rim of granulomas have been shown not to be in direct contact with infected macrophages, which impairs a proper control (15–17).

Our goal in this study was to shed light on the immune microenvironments of Mtb and the local diversity of Mtb-induced lesions in connection to their histopathology. For this purpose, we developed two automated identification pipelines to connect the localization of Mtb bacteria with immune transcripts in mouse lung tissue. Our investigation was divided into two parts: the connection of immune transcripts to single Mtb bacteria throughout the tissue in order to understand the proximal immune response to Mtb, and to bacteria-enriched clusters of structured or non-structured granulomas in order to understand the immune response towards larger infected areas.

The development of *in situ* sequencing (ISS), a spatial transcriptomics method, had a major role in advancing our ability to integrate functional and spatial information in biological studies (18, 19). While keeping the tissue architecture intact, ISS allows the detection of mRNA in tissue sections in a targeted approach. ISS is based on rolling-circle amplification (RCA) of padlock probes and was performed using sequencing-by-ligation chemistry. To complement the spatial distribution of detected immune transcripts and interaction networks in the TB mouse lung, we stained the same tissue for Mtb followed by staining for hematoxylin and eosin for histopathology annotation (13). In a previous approach, a cell-profiler-based pipeline enabled us to correlate the bacteria-staining with ISS transcripts, indicating the enriched presence of activated macrophages at subcellular distances to Mtb bacteria 8 weeks post infection of C57BL/6 mice (13). Here, we improved the detection of single bacteria in the tissue by machine-based learning, and additionally, we achieved an automated annotation of bacteria clusters.

Applying our bacteria-guided analysis of ISS data at different time points after infection in the lung tissue of C57BL/6 mice and in C3HeB/FeJ mice, we observed heterogenic bacteria–immune cell associations. C57BL/6 lungs showed enriched macrophage and activation markers in close proximity to bacteria that increased over time. In contrast, the immune environment in the lungs of Mtb-infected C3HeB/FeJ mice was dependent on the cluster size: small bacteria clusters showed a higher enrichment of innate immune activation transcripts in contrast to organized bigger clusters. Thus, these tools allowed us to better understand the diversity in immune cell composition activation of the Mtb tissue microenvironment.

## Methods

### Ethics Statement

The study was performed under the approval of the Stockholm North Ethical Committee on Animal Experiments (permit number N397/13 and N487/11). The animals were euthanized using cervical dislocation.

### Mice and Infection Assay

C57BL/6 mice were purchased from Janvier labs. C3HeB/FeJ mice were received from Igor Kramnik (BU, Boston, MA). All mice were housed and handled at the Astrid Fagreus Laboratory, Karolinska Institutet, Stockholm, under specific pathogen-free conditions and according to directives and guidelines of the Swedish Board of Agriculture, the Swedish Animal Protection Agency and the Karolinska Institutet. For the infection assay, mice were infected with 150–200 *Mycobacterium tuberculosis* (Mtb) Harlingen strain by aerosol using a nose-only exposure unit (In-tox Products). At the indicated time after infection, mice were euthanized and lungs were extracted and fixed in 4% buffered paraformaldehyde for 24 h. Fixed left lungs were paraffin-embedded. From each lung sample, 8-μm sections (along the long axis of the lobe) were obtained and stored at −80°C. Slides were paraffin-removed and dehydrated directly before further processing.

### *In Situ* Sequencing (ISS)

ISS was performed as described in Carow et al. (13, 19). After deparaffinization and digestion with pepsin 100 μg/ml in 0.1 M HCl for 20 min at 37°C, sections were washed with dH_2_O and PBS and then dehydrated through ethanol series. Hybridization chambers were mounted on top of the samples (SecureSeal Hybridization chambers, Grace Bio-Labs). Reverse transcription of mRNA was performed overnight at 37°C with the use of random decamer primers and specific primers that partially overlap with the sequence of the padlock probes in a mix containing 5 μM random decamers, 300 μM specific primers (IDT, Leuven, Belgium), 0.2 μg/μl BSA (NEB), 1 mM dNTPs (Blirt), 20 U/μl TranscriptMe reverse transcriptase (Blirt), and 1 U/μl RiboLock RNase Inhibitor (Blirt) in the TranscriptMe reaction buffer. The newly synthesized cDNA was crosslinked with 4% paraformaldehyde at room temperature for 45 min followed by degradation of mRNA and padlock probe hybridization and ligation performed in a mix containing 1× Tth buffer (20 mM Tris-HCl, pH 8.3, 25 mM KCl, 10 mM MgCl_2_, 0.5 mM NAD, and 0.01% Triton X-100), 10 nM of each padlock probe, 0.2 μg/μl BSA, 0.5 U/μl Tth ligase (Blirt), 0.4 U/μl RNase H (Blirt), 50 mM KCl, and 20% formamide (Sigma). Samples were incubated at 37°C for 30 min and then 45°C for 45 min. Multiple padlock probes were designed for each of the 36 transcripts of interest. Each padlock probe set for a target gene contains a unique four-base barcode, which is used for identification of target genes. Rolling circle amplification mix included 1 U/μl phi29 (Monserate Biotechnology), 0.4 U/μl ExoI (Thermo Scientific), 0.25 mM dNTPs, and 5% glycerol in phi29 buffer [50 mM Tris-HCl, pH 7.5, 10 mM MgCl_2_, and 10 mM (NH4)_2_SO_4_]. The samples were incubated at 37°C for 5 h and then at 30°C overnight, generating the rolling cycle product (RCP) for detection.

Sections were then washed and 0.5 μM Alexa750 anchor probe was hybridized to the amplification products for 45 min at room temperature in the dark in a mix containing 2×SSC and 20% formamide. For sequencing by ligation, 0.1 μM of each of the four interrogation probes conjugated with corresponding fluorophores (FITC, Cy3, Cy5, or TexasRed) were incubated for 1 h at room temperature in the dark with a mix containing 0.1 U/μl T4 ligase (Blirt), 1× T4 ligase buffer, 0.2 mg/ml BSA, 1 mM ATP (Thermo Scientific), and 100 ng/ml DAPI. After PBS washes, sections were dehydrated through ethanol series and air dried. Cover slips were mounted with SlowFade™ Gold anti-fade reagent (Invitrogen). Imaging was performed in a Axio Imager Z2 epifluorescence microscope (Zeiss) with 20× objective by acquiring Z-stacks of overlapping tiles that together cover the tissue section (10% overlap).

After imaging, the samples were prepared for the next sequencing cycle. For the removal of the interrogation probes, the samples were treated with 0.02 U/μl UNG (Thermo scientific) and 0.2 μg/ml BSA in UNG treating buffer for 15 min and washed twice with DEPC-PBS-T. Three washing steps with 100% formamide for 3 min followed. After washing with DEPC-PBS-Tw, the anchor hybridization, ligation, and imaging processes were repeated for the next base. A full list of padlock probes, reverse transcription primers, and sequencing-by-ligation oligonucleotides is available in the work of Carow et al. (13).

### Auramine–Rhodamine T Staining

After ISS, the cover slips were removed from the glass slides, the tissue sections were washed in PBS, and TB Auramine–Rhodamine T (AR) staining was performed using the TB Fluorescent staining kit T (BD). Briefly, the tissue sections were incubated at 37°C for 15 min in pre-warmed TB AR (BD), washed with tap water, and decolorized for 4 min at room temperature with Decolorizer. After being washed with tap water, the tissue sections were stained with DAPI for 15 min in room temperature, washed with tap water, and air dried, and cover slips were mounted with SlowFadeTM Gold anti-fade reagent (Invitrogen). Imaging was performed in an Axio Imager Z2 epifluorescence microscope (Zeiss) with 20× objective by acquiring Z-stacks of overlapping tiles that together cover the tissue section (10% overlap).

### ISS Image Analysis

The z-stacks of acquired images were merged to a single image using the maximum-intensity projection (MIP) in the Zeiss ZEN software. The resulting images were stitched into a single image containing the entire scanned area. The images from the four sequencing rounds were aligned based on the DAPI staining. For the analysis of the aligned images, a CellProfiler (v2.1.1) automated pipeline was used (Github repository, Blobidentification_NucleiCounting), which applied ImageJ plugins for image registration. A manual threshold for identification of primary objects was set between 0.0015 and 0.0020. The general stain from RCPs (anchor probe) was used for saving *x* and *y* coordinates and the fluorescence intensities for each RCP for each of the four barcode bases were saved in a.csv file and decoded using a MATLAB script (Github repository, InSituSequencing_1). For each RCP and sequencing round, the base with the highest intensity was assigned to the corresponding RCP and a fixed quality threshold of 0.35 that was defined as the maximum signal divided by the sum of all signals for that base was applied.

For signal visualization the InSituSequencing_1 MATLAB script was used to plot the transcripts on DAPI, hematoxylin–eosin (HE), or AR staining images.

### Identification of Single Bacteria and Surrounding Transcripts

AR staining images were used for the single bacteria identification. Fluorescence from FITC channel was subtracted from the Cy3 channel to reduce background fluorescence (ImageSubstraction MATLAB script) and the images were tiled with InSituSequencing_V3 MATLAB script (both in Github repository). The tiled images were used as input for a Cell profiler pipeline (IdentifyImagedBacteria) (v 4.0.6) for the identification of all imaged objects (Github repository). The intensity range for the input images was set between 0 and 0.5 except for sections in which we observed oversegmentation of background and then the threshold was set to 0.008/0.015–0.5. The selected thresholding method was Otsu 3-classes, which calculates the threshold separating the three classes of pixels (foreground, background, and middle intensity) by minimizing the variance within each class. Threshold correction factor was set to 0.4 and the size of the smoothing filter was set to 8. The generated .properties file and training set were used as input for the Cell profiler-Analyst (v 2.2) to classify single bacteria, bacterial clumps (when few bacteria are very close to each other and create one identifiable object), and background. A.txt file was generated containing 20 rules for optimal object classification (rules_Mixed_20, Github repository).

The obtained object classification rules were used in a final Cell profiler (v 4.0.6) pipeline with tiled AR images as input. The parameters used were similar to the Cell Profiler pipeline for the identification of all the objects (Github repository, FinalSingleBacteria).

The generated .csv files with the *x* and *y* coordinates of identified single bacteria and bacterial clumps were merged and used as input for the RelateBacteriaAndGene MATLAB script together with the (details) .csv file obtained from the MATLAB decoding step that contains the *x* and *y* coordinates of the ISS-identified transcripts from the same tissue (Github repository). From this analysis, a QT_details_ParentBact.csv file was obtained, which contained the distance of each transcript to its closest bacteria. Transcripts within given bacteria distances were extracted, and the frequency of each immune transcript among all immune transcripts of the given distance was used for analysis and normalized to the frequency of the transcript in the total slide.


$$\text{Frequency ratio}=\frac{\%\;\text{of transcript}\;X\;\text{in distance}\;Y}{\%\;\text{of transcript}\;X\;\text{in total slide}}$$

### Identification of Bacterial Clusters and Surrounding Transcripts

AR staining images were re-sized to 20% of the original image and used for the bacterial cluster identification. The re-sizing allowed the process of one (smaller size) image per tissue section, avoiding the tiling that is needed for processing original files and thus not interrupting the recognition of fluorescence signal from cluster areas. Subtracted images created as described above were processed with an automated MATLAB script (Github repository, BacterialClusters_wGradientOutput_Areas) for the cluster identification and gradient border assignment. For the bacterial cluster identification, a manual threshold between 78% and 95% was set for optimal identification depending on how widespread the staining for each tissue was. A threshold of 90% means the top 10% pixels are considered as foreground. The identified clusters were numbered and clusters with area smaller than 7,000 pixels^2^ were excluded from further analysis. A gradient of selected size 300 pixels (97,5 μm) was drawn around the selected clusters. We were able to identify the transcripts reads falling in each cluster area or surrounding gradient by using the *x* and *y* coordinates of each identified transcript, which was produced during decoding with the InSituSequencing_1 MATLAB script. Finally, the density of each transcript as the number of transcript reads divided by cluster area or gradient step area was used for further analysis. For comparisons between sections, the transcript density was normalized by a performance factor as the ratio of total number of identified immune transcripts and total section area.


$$\text{density ratio}=\frac{\frac{\text{selected transcript number }\left(\text{ROI}\right)}{\text{area }\left(\text{ROI}\right)}}{\frac{\text{total transcript number }\left(\text{section}\right)}{\text{area section}}}$$

### Heatmap and Principal Component Analysis

Normalized transcript reads or frequencies were uploaded to ClustVis, a web tool for visualizing clustering of multivariate data using heatmaps and principal component analysis (https://biit.cs.ut.ee/clustvis/). ClustVis calculates principal components using one of the methods in the pcaMethods R package and plots heatmaps using the heatmap R package (version 0.7.7).

### Statistics

Student’s *t*-test and ANOVA were used as indicated with *p* < 0.05 marked as significant (*), *p* < 0.01 (**), *p* < 0.001 (***), and *p* < 0.0001 (****).

### Github Repository

All relevant scripts for ISS image analysis, the single bacteria identification, and the bacterial cluster identification are available at https://github.com/Moldia/Tuberculosis-bacterial-analysis.

## Results

### ISS Identifies Expected Patterns of Immune Transcripts in Tuberculous Lung Granulomas and Allows the Analysis of Mtb Immune Environments

In this study, we extended the analysis of the distribution of 33 immune and 3 control transcripts in lung sections of Mtb-infected mice (13) with the focus of describing the immune environment of Mtb bacteria in different mouse infection models and at different time points post infection. We have generated new datasets in order to compare two different Mtb mouse models, C57BL/6 and C3HeB/FeJ, since they show different pathologies after Mtb infection: the former does not develop necrotic lesions after Mtb infection, while the latter is susceptible to Mtb and shows necrotizing granulomas (20).

**Figure 1:**
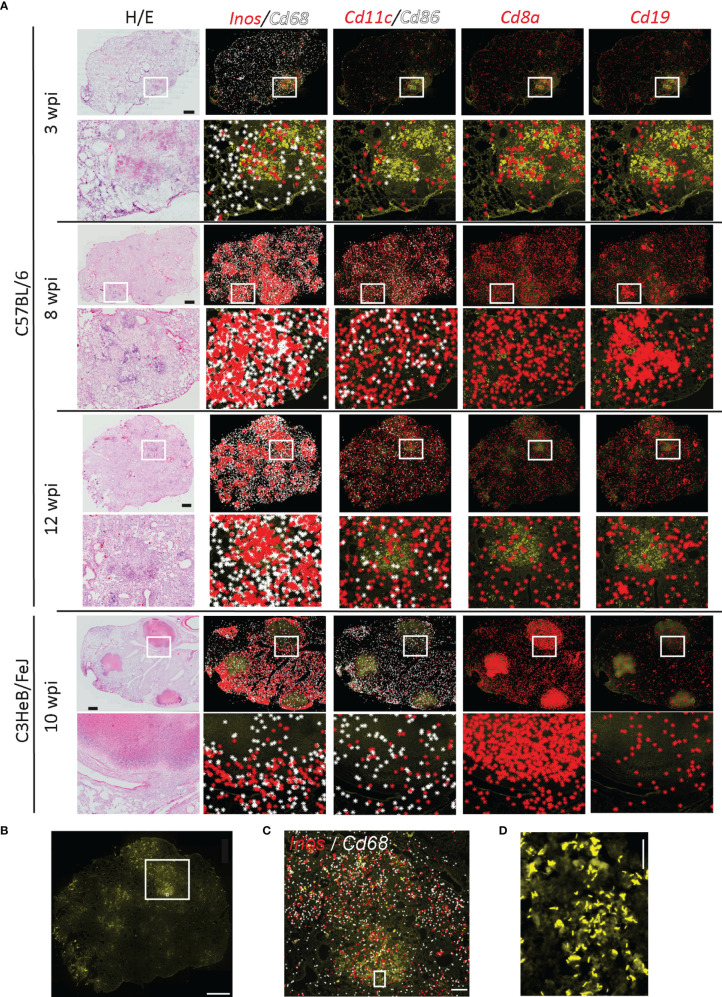
Immune environments of *Mycobacterium tuberculosis* bacteria. Localization of immune transcripts in tuberculous lung granulomas. **(A)** Fixed lung tissue sections of C57BL/6 and C3HeB/FeJ mice at the indicated weeks post Mtb infection (wpi) were analyzed by ISS and stained for DAPI, Auramine–Rhodamine T (AR), and hematoxylin–eosin (HE). ISS signals for indicated transcripts were plotted on AR-stained image as background. One representative image out of the three sections per condition is displayed. Scale bar: 1,000 μm. **(B)** Image of AR staining of C57BL/6 lung section 12 wpi is displayed with a scale bar of 1,000 μm. Rectangular regions for zoom-ins are indicated. **(C)** *Inos* and *Cd68* transcripts plotted on AR image (zoom-in of B). Scale bar: 200 μm. **(D)** Magnification of AR image in B and C is shown for visualization of single bacteria and bacterial clumps. Scale bar: 20 μm.

By aligning the generated transcript signals to the HE staining of the analyzed lung tissue section, we found the predicted patterns of immune transcripts and that our control *Cc10* transcript (coding for Clara cell 10-kDa protein) localized as expected in airway epithelium (**Figure 1A** and **Supplementary Figure 1A**). For base identification in the barcode reading of the sequencing rounds, we chose a quality score of 0.35, defined as the maximum signal divided by the sum of all signals for that base. We further identified comparable expression patterns and a strong linear correlation (*r* ^2^ = 0.97) when using different ISS quality thresholds (**Supplementary Figures 1B, C**).

By exploring the distribution of transcripts to identify major immune cell subsets, we found that transcripts of macrophage-indicating *Cd68* and of *inducible nitric oxide synthase-2* (*Inos*) that are expressed in activated macrophages increased from 3 weeks post Mtb infection (wpi) to 8 and 12 wpi. These transcripts were absent in encapsulated granulomas from C3HeB/FeJ mice at 10 wpi (**Figure 1A**). *Cd11c* and *Cd86* transcripts expressed by antigen-presenting cells showed a scattered distribution (**Figure 1A**). However, the frequency of both *Cd11c* and *Cd86* significantly increased at 8 and 12 weeks as compared to 3 wpi (**Supplementary Figure 1D**). As expected, frequency and occurrence of T cell-related transcripts like *Cd8a* were elevated after 3 wpi. In C57BL/6, the distribution was even across the lung tissue, whereas in C3HeB/FeJ mice, they localized mainly to organized granuloma structures. *Cd19* transcripts localized to lymphoid-rich histology structures (**Figure 1A**). These potential B-cell follicles formed at the later time points in C57BL/6 lungs, but were reduced in the lungs of C3HeB/FeJ mice, confirming our previously published results (13) in this new C57BL/6 ISS dataset.

To determine immune transcripts that localized in the proximity of Mtb, we performed AR staining for the analyzed sections (**Figure 1B**). Bacteria accumulated in high-density regions of *Cd68* and *Inos* transcripts, as reported previously (13) (**Figure 1C**). As we were able to locate single bacteria and immune transcripts generated by ISS (**Figures 1C, D**), we aimed to describe the immune molecules in the proximal to single bacteria and bacteria accumulated in lesions by developing two different automated pipelines.

### Single Bacteria Identification Indicates Robust *Inos* Expression in Mtb-Infected Cells in C57BL/6 but not in C3HeB/FeJ Lungs

As we were able to locate single bacteria and immune transcripts generated by ISS (**Figures 1C, D**), we aimed to describe the immune molecules in the proximal to single bacteria and bacteria accumulated in lesions by developing two different automated pipelines.

**Figure 2:**
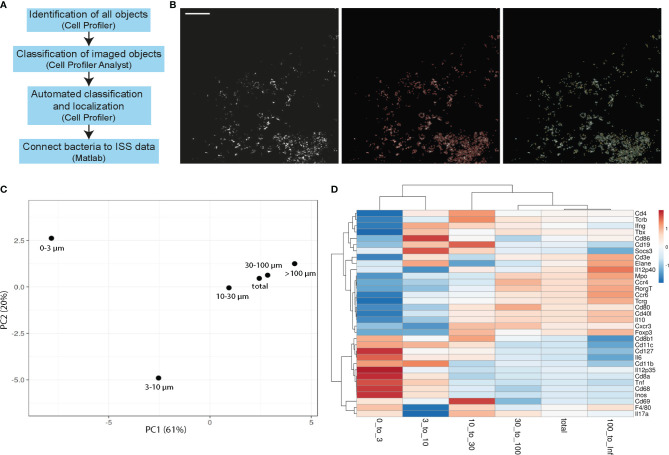
Single bacteria identification and their immune environment per distance. **(A)** Workflow scheme of single bacteria identification pipeline development and connection to ISS data. **(B)** Example of tiled AR image that is used as input into the single bacteria identification (final FinalSingleBacteria) pipeline is shown in the left panel. Automated detection of all imaged objects is annotated in red in the middle panel and identified single bacteria (yellow) and bacteria clumps (green) are in the right panel. Scale bar: 100 μm. **(C, D)** Principal component analysis **(C)** and heatmaps **(D)** of immune transcript frequencies at different distances from identified bacteria (0–3 μm, 3–10 μm, 10–30 μm, 30–100 μm, >100 μm) and the frequency taken over the total section are displayed for one representative C57BL/6 lung section at 3 wpi.

To objectively and automatically detect the Mtb bacteria in the tissue, we applied a machine learning approach to identify all bacteria in lung sections, to exclude background noise, and to connect the bacteria coordinates to ISS data (**Figure 2A**). As input, we used the Cy3 channel of AR images that had been subtracted by the FITC channel of the same image to reduce the impact of autofluorescence. In a first Cell Profiler pipeline, objects in tiled images were detected. These objects were then loaded in Cell Profiler analyst and manually sorted for a correct classification into single bacteria, clumps (2–4 bacteria that are not distinguishable), and background. Using the fast gentle boosting algorithm in Cell Profiler analyst, we retrieved 20 rules based on object shape and intensity that were of more than 88% accuracy to correctly classify unknown objects (**Supplementary Figure 2A**). Implementing the identified rules in the initial Cell Profiler pipeline (Github repository, FinalSingleBacteria) allowed the detection of objects, retrieving the identification and coordinates for single bacteria and clumps of bacteria (**Figure 2B**).

Next, we used a MATLAB script (Github repository, RelateBacteriaAndGene) to measure the distance of each transcript to the nearest bacteria, and found distinct frequencies of immune transcripts at different distances from bacteria (**Figures 2C, D**). Results for single bacteria and bacteria clumps were similar, and therefore pooled data were analyzed (**Supplementary Figure 2B**). In a C57BL/6 lung section, 3 wpi showed the most distinct transcript frequencies of innate immune transcripts (e.g., *Cd11b, Cd11c, Cd68, Inos, Il12p35*, and *Il6*) at subcellular distances (0–3 µm and 3–10 µm) from Mtb, which we interpret as high expression within the infected cells or in a cell neighboring an infected cell (**Figures 2C, D**). By computing the ratio of a transcript frequency at a given distance to the transcript frequency across the whole slide, we estimate over/underrepresentation of a given transcript in the selected distance. As transcript frequencies varied between the time points and models, only comparisons between the distances within one time point/model were performed (**Supplementary Figure 1D**).

**Figure 3:**
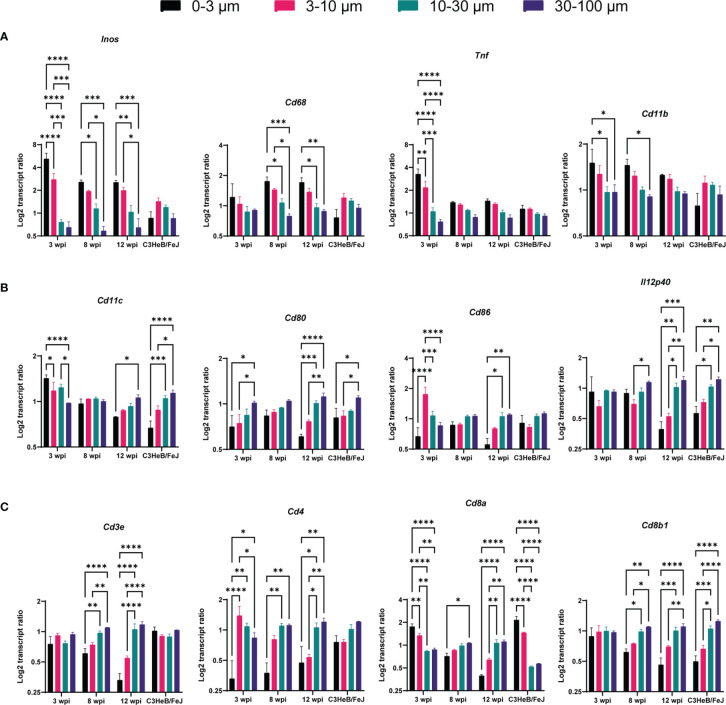
Single bacteria identification and their immune environment per transcript. Transcript frequency ratios for indicated distances to bacteria were identified in C57BL/6 mice 3, 8, and 12 wpi and in C3HeB/FeJ mice at 10 wpi. Transcript frequencies for **(A)** *Inos, Cd68*, *Tnf*, and Cd11b; **(B)** *Cd11c*, *Cd80*, *Cd86*, and *Il12p40*; and **(C)** *Cd3e*, *Cd4*, *Cd8a*, and *Cd8b1* are displayed. The transcript frequency ratios were calculated as the % of a transcript at a selected distance divided to the % of this transcript in the total slide. The mean of log2 ratio ± SEM of indicated transcripts in three sections per condition are shown (ANOVA, * for *p* < 0.05, ** for *p* < 0.01, *** for *p* < 0.001, and **** for *p* < 0.0001).

A significant increase of transcripts indicating activated macrophages was found at subcellular distances compared to longer distances in C57BL/6 lungs, indicated by *Inos* expression at all time points and for *Cd68, Cd11b*, and *Tnf* at some time points (**Figure 3A**), analyzing three sections per condition. In contrast, applying this analysis of single bacteria surroundings in C3HeB/FeJ lungs at 10 wpi did not show any preferential expression pattern of macrophage and activation markers. The elevated *Cd11c* transcript ratio at subcellular distances at 3 wpi could indicate the infection of *Cd11c*-expressing cells as monocytes, alveolar macrophages, or dendritic cells (DCs) by Mtb (**Figure 3B**). However, at 8 wpi, no differences between subcellular and longer distances were observed. At 12 wpi, an opposite pattern was found with a preferential *Cd11c* expression at longer distances. Transcripts for co-stimulatory molecules *Cd80* and *Cd86* involved in antigen presentation and T-cell activation as well as Th1 differentiation cytokine *Il12p40* similarly displayed an increase with distance at 12 wpi. C3HeB/FeJ lungs showed a similar pattern with increased DC-related transcript ratios for longer distances to identified bacteria (**Figure 3B**). As expected, T cell-indicating transcripts were preferentially found for distances >10 µm (**Figure 3C**). However, the transcript ratio for *Cd8a* was increased at subcellular distances to Mtb in lungs of C57BL/6 mice at 3 wpi and of C3HeB/FeJ mice.

### Bacteria Cluster Analysis Highlights Spatial and Temporal Changes Upon Infection in C57BL/6 Lungs

The single bacteria identification analysis gave a robust overview of the Mtb immune environment in the analyzed tissue; however, it might be a more relevant tool for studying the disease development in the early stages upon infection. That is, because in early stages of the disease, the bacteria are more spread throughout the tissue, compared to later time points when Mtb aggregates in lesions creating distinct microenvironments. Our above pipeline described an average immune environment around single bacteria or small clumps without the ability to display the environment’s heterogeneity. Thus, we aimed to automatically identify individual bacteria clusters corresponding to tuberculous lung lesions to analyze their associated immunogenic heterogeneity.

**Figure 4:**
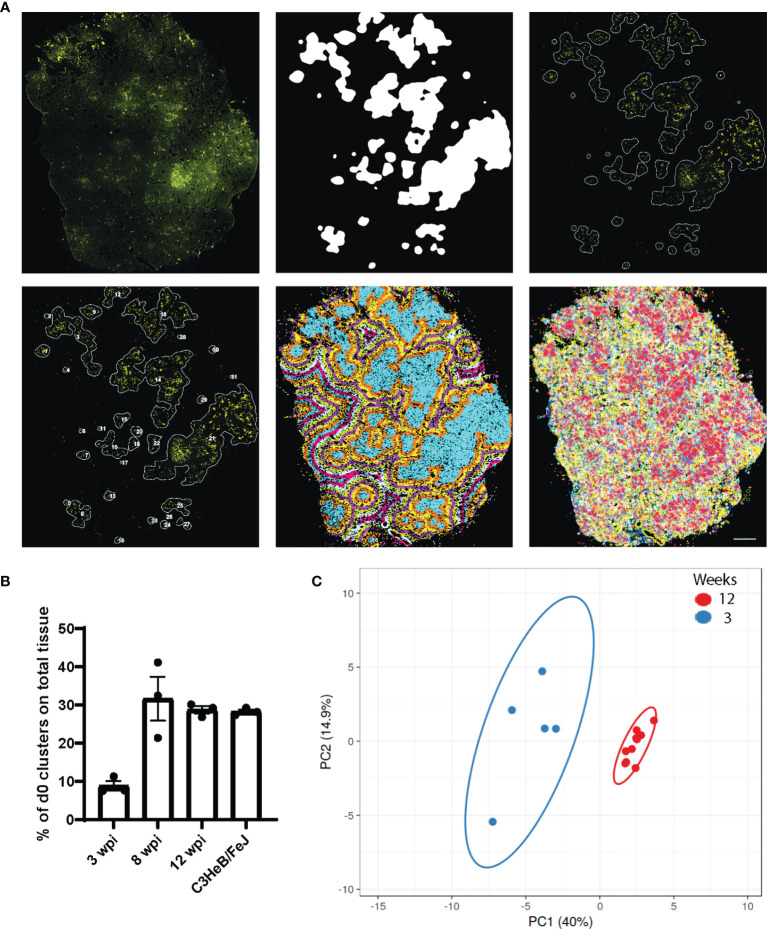
Bacteria clusters and their immune pattern in C57BL/6 mice. **(A)** Exemplary input and output of bacteria cluster pipeline for one C57BL/6 section at 12 wpi shows the AR image (top left) and cluster identification on the 20% subtracted image (top middle, top right), based on AR staining signal intensity. Lower panel displays cluster labeling for individual cluster analysis (bottom left), the applied 100 μm gradient around identified clusters (bottom middle), and plotted immune transcripts (bottom right). Scale bar: 1,000 μm. **(B)** Percentage of tissue area occupied by bacteria cluster (d0) for C57BL/6 lung sections at 3, 8, and 12 wpi and for C3HeB/FeJ lung sections at 10 wpi is displayed. Mean frequencies ± SEM for three sections per condition are shown. **(C)** Principal component analysis displays the density ratio of immune transcripts in bacteria clusters identified in a representative section at 3 (blue, *n* = 5) and 12 (red, *n* = 10) wpi in C57BL/6 mice. The ellipse around the clusters indicates the area in which 95% of the samples would be located based on gaussian distribution.

For this purpose, we developed a MATLAB script that used a 20% image of the Auramine staining (Cy3 channel subtracted by FITC channel as above) as this enabled us to analyze the whole tissue section without dividing images by image tiling. The bacteria cluster identification was based on the intensity of the AR staining applying a top-hat filter, smoothening and thresholding of the image using parameters that consistently gave a satisfactory separation (Github repository, BacterialClusters_wGradientOutput_with_areas) (**Figure 4A**; **Supplementary Figures 3A, B**). The identified clusters were connected to the ISS coordinates resulting in extracted reads from each assigned cluster. To study areas within distance to the cluster, we additionally extracted ISS reads from areas in 100-µm steps starting from the cluster borders. As the ISS assay performance varies between sections, we normalized the transcript density per cluster to the density of immune transcripts per slide, which allowed the comparison between distances and time points.

**Figure 5:**
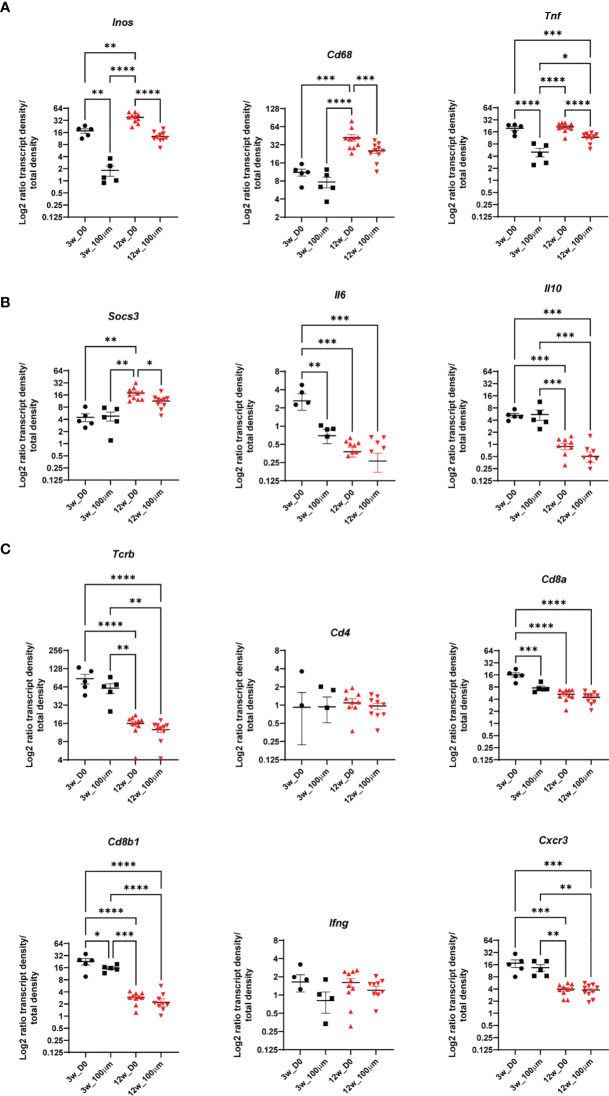
The immune environment of bacteria clusters and bacteria cluster surrounding areas in C57BL/6 mice. Comparisons of density ratios for indicated transcripts from selected identified bacteria clusters (d0) are displayed with the addition of density ratios of the 100-μm surrounding (d100) of the bacteria clusters for one representative C57BL/6 lung section at 3 (black) and 12 (red) wpi. Transcript density ratios are displayed **(A)** *Inos*, *Cd68* and *Tnf*; **(B)** *Socs3, Il6*, and *Il10* cytokines; and **(C)** *Tcrb*, *Cd4*, *Cd8a*, *Cd8b1*, *Ifng*, and *Cxcr3*. Mean density ratios ± SEM and individual values are shown, and significant differences are indicated (ANOVA, * for *p* < 0.05, ** for *p* < 0.01, *** for *p* < 0.001, and **** for *p* < 0.0001).

The percentage of tissue area that was occupied by bacteria clusters increased from 10% at 3 wpi to around 30% at 8 and 12 wpi, showing the dissemination of bacteria in the lung tissue over time (**Figure 4B**). We compared the ratios of all expressed transcripts at 3 and 12 wpi in C57BL/6 lungs in the bacteria clusters containing the highest transcript numbers to ensure data robustness (5 highest at 3 wpi, 10 highest at 10 wpi). This analysis showed that any cluster from 3 wpi was distinct from 12 wpi, although some transcript ratios could overlap at the individual cluster level (**Figures 4C**, **5**). The range of ratios within one section could reflect the immune heterogeneity of bacteria clusters. Only 3 transcripts (*Inos*, *Cd68*, and *Socs3*) increased over time within the analyzed bacteria clusters (**Figures 5A, B**). We expected that the most relevant immune responses to influence the pathogen–host interaction would occur within the identified cluster and within the surrounding 100-µm radius (**Supplementary Figure 4A**). In line with our single bacteria identification, innate immune response-associated transcripts (*Inos* and *Tnf)* showed a strong decrease with an increased distance to the bacteria cluster (**Figure 5A**). The increase of *Socs3* from 3 to 12 wpi within the bacteria cluster showed a preference for the bacteria cluster and lower levels in the cluster-surrounding 100 μm (**Figure 5B**). SOCS3 is a negative feedback regulator of cytokine and growth hormone signaling and has been shown to regulate the immune response against Mtb within several immune cell types (21, 22). However, transcripts of possible SOCS3-inducing cytokines—*Il6* and *Il10*—did not follow the same pattern as *Socs3* (**Figure 5B**). Most T cell-related transcript density ratios decreased from 3 to 12 wpi, which was unexpected (**Figure 5C**). Importantly, no differences between transcript ratios within the cluster and its 100-μm gradient could be detected at 12 wpi, indicating an even distribution of T cells in the tissue.

### Bacteria Clusters and Their Immune Environments in C3HeB/FeJ Mice Show High Molecular and Histopathological Heterogeneity

**Figure 6:**
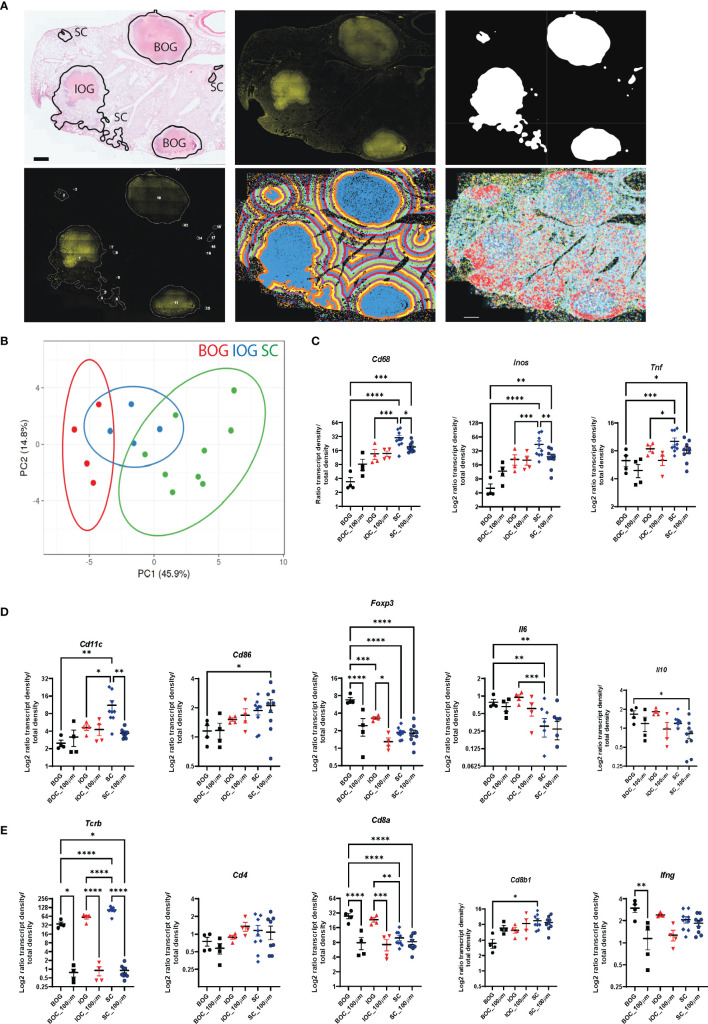
Bacteria cluster types and their immune environment in C3HeB/FeJ mice at d(0) and d(100). **(A)** Annotation of cluster types shown on HE image to show the underlying histopathology for one representative C3HeB/FeJ section at 10 wpi (top panel, left). Classification into BOG, big organized granulomas, IOG, intermediate organized granulomas (less pronounced encapsulated structure), and SC, small cluster. Exemplary input and output of bacteria cluster pipeline with AR image (top middle) and cluster identification on the 20% subtracted image (top right). Lower panel displays cluster labeling for individual cluster analysis (left), the applied 100-μm gradient around identified clusters (middle), and plotted immune transcripts (right). Scale bar: 1,000 μm. **(B)** Principal component analysis displays the density ratio of immune transcripts in bacteria clusters identified across 3 sections for BOG (red, *n* = 4) IOG (blue, *n* = 4), and SC (green, *n* = 9). The ellipse around the clusters indicates the area in which 95% of the samples would be located based on gaussian distribution. **(C–E)** Density ratios for indicated transcripts of the same clusters as in **(B)** are displayed. The mean of transcript densities in the bacteria cluster (d0) and in the 100-μm (d100) surrounding area was calculated. Displayed transcripts in **(A)** *Cd68, Inos*, and *Tnf*; **(B)** *Cd11c, Cd86, Foxp3, Il6*, and *Il10*; and **(C)** *Tcrb, Cd4, Cd8a, Cd8b1*, and *Ifng*. Mean density ratios ± SEM and individual values are shown, and significant differences are indicated (ANOVA, * for *p* < 0.05, ** for *p* < 0.01, *** for *p* < 0.001, and **** for *p* < 0.0001).

Next, we applied our bacteria cluster identification script to find and describe bacteria clusters in Mtb-infected lung sections of C3HeB/FeJ mice. After automated cluster identification, we manually grouped the clusters based on their underlying histopathology into three different lesion types that we called BOG (big, organized granulomas), IOG (intermediate, organized granulomas), and SC (small clusters) (**Figure 6A** and **Supplementary Figure 5A**). BOG and IOG bacteria clusters showed a round structure with a center of necrotic cells surrounded by a lymphocytic rim, whereas SC bacteria clusters were of uneven structure, mainly consisting of epithelioid cells (**Figure 6A** and **Supplementary Figure 5A**). Comparing the transcript ratios of all immune transcripts contained in the cluster types, we observed a clear distinction between BOG and SC, with IOG showing overlap within both groups (**Figure 6B** and **Supplementary Figure 5B**).

The grouping of bacterial clusters allowed a refinement of the single bacteria analysis that had shown lower transcript levels associated with innate-mediated protection compared to C57BL/6 mice. We found reduced levels of macrophage and dendritic cell-associated transcripts (*Cd68, Inos, Tnf*, and *Cd11c*) in BOG compared to SC (**Figures 6C, D**). T cell-associated transcripts showed higher expression levels of *Tcrb* and *Cd8b1* in SC and increased *Cd8a*, *Ifng*, and *Foxp3* levels in BOG (**Figures 6D, E**). *Cd4* transcript levels were comparable between BOG, IOC, and SC (**Figure 6E**). Most of the differences in transcript levels were very localized as they were only present within the bacteria clusters and transcript patterns of BOG and SC overlapped when applying the 100-μm radius around the identified clusters (**Figures 6C–E**; **Supplementary Figures 4B**, **5B**).

### Similarities in Expression Patterns of SC in C3HeB/FeJ Mice With C57BL/6 Lesions Despite Genetic Differences

**Figure 7:**
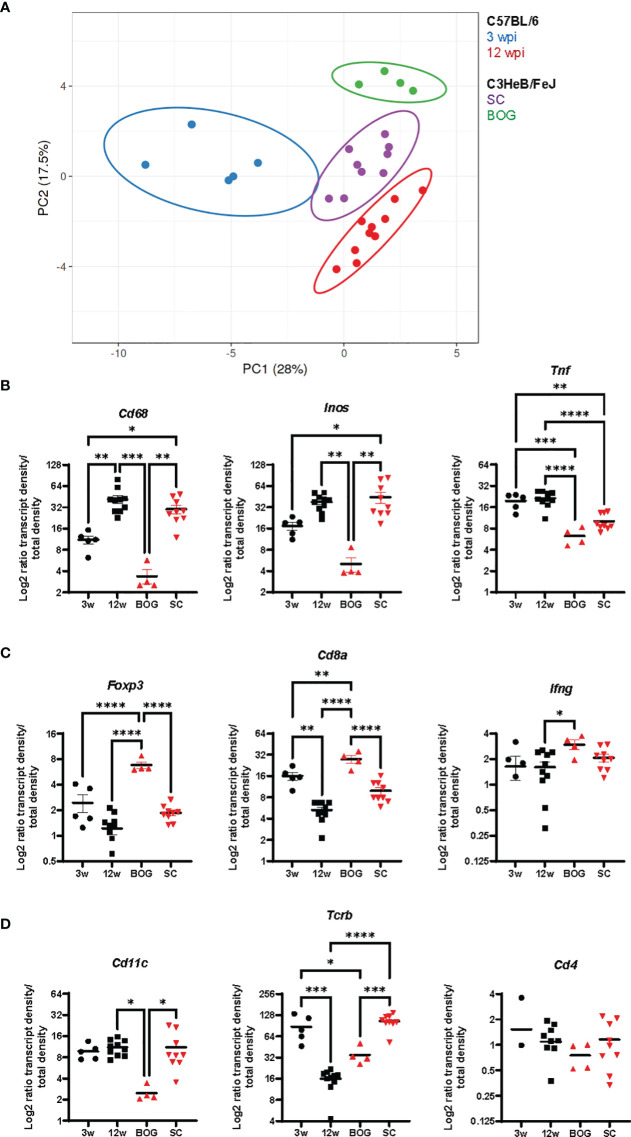
Bacteria cluster comparison of C57BL/6 and C3HeB/FeJ mice. **(A)** Principal component analysis of the density ratio of immune transcripts in bacteria clusters of C57BL/6 and C3HeB/FeJ mice is shown. Clusters were identified as described for **Figures 5**, **6** with 3 wpi C57BL/6 (blue, *n* = 5), 12 wpi C57BL/6 (red, *n* = 10), C3HeB/FeJ SC (purple, *n* = 9), and BOG C3HeB/FeJ (green *n* = 4). The ellipse around the clusters indicates the area in which 95% of the samples would be located based on gaussian distribution. **(B–D)** Density ratios for indicated transcripts of the same clusters as in A are displayed: **(A)** *Cd68, Inos*, and *Tnf*; **(B)** *Foxp3, Cd8a*, and *Ifng*; **(C)** *Cd11c, Tcrb*, and *Cd4*. Mean density ratios ± SEM and individual values are shown, and significant differences are indicated (ANOVA, * for *p* < 0.05, ** for *p* < 0.01, *** for *p* < 0.001, and **** for *p* < 0.0001).

Finally, to illustrate differences in localized immune environments of Mtb in lungs of C57BL/6 and C3HeB/FeJ mice considering cluster types and time points after infection, we compared expression patterns in the bacteria cluster of C57BL/6 mice at 3 and 12 wpi with BOG and SC in C3HeB/FeJ mice at 10 wpi (**Supplementary Figure 6**). A principal component analysis of normalized transcript densities placed the SC of C3HeB/FeJ mice between 3 and 12 wpi bacteria clusters of C57BL/6 mice (**Figure 7A**). BOG clustered distinct from SC and from C57BL/6 clusters. The expression levels of *Cd68, Inos*, and *Tnf* were reduced in BOG clusters not only compared to SC but also in comparison to clusters in early and late time points in Mtb-infected C57BL/6 lungs (**Figure 7B**). In contrast, the BOG characteristic elevated *Cd8a* and *Foxp3* transcripts were found at increased levels compared to SC but also in comparison to 3 and 12 wpi bacteria clusters of C57BL/6 mice (**Figure 7C**). Altogether, this “cross-comparison” further emphasizes the compartmentalization in two different bacteria environments in C3HeB/FeJ mice.

## Discussion

The here-presented data successfully resolve the spatial interaction of immune transcripts representing the host’s immune response and Mtb bacteria in different mouse models and time points after infection. Using two complementary pipelines, our work describes the subsequent activation of innate immunity at subcellular distances to Mtb over time in lungs of infected C57BL/6 mice without the observation of a spatial organization of T-cell responses. Transcripts indicating antigen presentation (*Cd11c, Cd80*, and *Cd86*) are enriched in non-infected cells at later stages of infection. In C3HeB/FeJ mice, we found that the immune environment differed between lesion types: in small clusters that resemble the C57BL/6 clusters in their expression pattern, macrophage transcripts like *Cd68* and *Inos* were enriched, whereas in organized granulomas, we observed *Cd8a, Tcrb*, and *Foxp3* enrichment.

### Spatial Analysis

Recent developments in the field of spatial transcriptomics and metagenomics have been focusing either on the identification of multiple microbial species *in situ* (23, 24) or on the identification and transcriptional analysis of selected microbial species (25). Other approaches have associated the local expression profile with the manually annotated histopathological features of the tissue or the expression of specific disease markers (26).

Immunological studies of the TB granuloma have applied several spatially resolving techniques like laser caption microscopy in combination with proteomics as well as multiplexed ion beam imaging by tune of flight (MIBI-TOF) on human tissue sections containing active TB granulomas to find that granuloma structure and immune cell function are connected (27, 28). McCaffrey et al. imaged 37 proteins in TB granulomas constructing an atlas identifying 19 cell subsets and 8 spatial microenvironments. Importantly, one identified microenvironment indicated immune dysfunction characterized by IFN-γ depletion and an enrichment for TGF-β, regulatory T cells, and IDO1- and PD-L1-expressing myeloid cells. Their analysis increases our understanding of granuloma immunology and suggests key events leading to active TB, which opens for new therapies. However, all the aforementioned approaches lack the association of the microbial presence with the local immune profile of the tissue.

Mtb is unique with regard to several aspects of pathogen–host interactions, the formation of granulomas, a non-sterilizing T cell-mediated immunity, and a bacterial persistence program as some characteristics. As one step in understanding local immune responses to Mtb, we present automated analysis pipelines to combine the localization of Mtb bacteria and immune transcripts, which allows the definition of differences in the environment that is proximal to bacteria. The single bacteria detection pipeline used allowed us to study the immune response locally in selected distances from Mtb in infected mouse lungs. This approach proved valuable in the early stages of infection and in sections of tissue where Mtb is spread or scarce, and where only frequencies of immune transcripts in given distances could be calculated (no normalization to area possible). In later stages of Mtb infection, the bacteria form clusters corresponding to histopathological lesions or tuberculous granulomas depending on the murine genetic background. With the cluster analysis, we were able to identify the bacteria clusters according to the intensity of the AR Mtb staining and the adjacent areas allowed to calculate the immune transcript density. Combining results gained from the single bacteria and the bacteria cluster analysis gave insights into different aspects of the occurring pathogen–host interactions. Regarding our results, the presentation of one sample per time point (in triplicate) limits the conclusions that can be drawn, but the robustness and multiplexity of the generated data still allow us to generate suggestions that may be evaluated in extended studies.

### Mtb and Innate Immunity

Mtb has developed mechanisms to escape the microbicidal mechanisms of macrophages and can instead survive and replicate within them (29). We found a high expression of *Cd68* and *Inos* at subcellular distances to bacteria as well as within bacteria clusters in C57BL/6 mice lungs. *Inos* and *Tnf* levels surrounding infected cells and clusters were also elevated. Similarly, we found elevated *Cd68* and *Inos* levels in small bacteria clusters (SC) of C3HeB/FeJ mice but not in their organized granulomas. No subcellular increase for those transcripts was found in the single bacteria analysis for C3HeB/FeJ mice, probably since the average Mtb surrounding represents a mixture of SC and BOG. The expression of *Inos* at subcellular levels is indicative of classically activated M1 macrophages upon infection of the cell. However, as no M2 markers were included in the panel, we cannot compare the M1/M2 differentiation for infected cells and the Mtb immune environment, which remains to be studied in the future.

*Inos* encodes for the enzyme producing bactericidal nitric oxide (NO) and is central in the innate defense against Mtb in mice, and its absence leads to mice that are highly susceptible to Mtb (30, 31). The frequency of *Inos* mRNA at subcellular distances to Mtb increased from 3 to 8 and 12 wpi as well as the lung area occupied by bacteria clusters. We found the highest expression of *Inos mRNA* within the infected cells but not in neighboring cells, excluding a direct transcriptional repression by Mtb. Despite its correct cellular location and high expression, there are several possibilities that may account for the insufficient Mtb control: Toxic NO levels may not have been reached due to limitation of the iNOS substrate L-Arginine or insufficient enzymatic activity of iNOS. Another possibility is that Mtb’s evasion mechanisms prevented the bacterial clearance. Mtb has been shown to produce proteins that repair NO-damaged bacterial proteins at the proteasome level (32–34) and inhibits the recruitment of the enzyme iNOS to the phagosome (35).

Another transcript density that increased over time in C57BL/6 bacteria clusters besides *Cd68* and *Inos* was *Socs3*. SOCS3 is a negative feedback regulator of cytokines of the IL-6 family, and several growth factors and hormones and SOCS3 in myeloid cells contribute to defense against Mtb (21, 22). As transcripts of its main inducing cytokines, IL-6 and IL-10 did not increase within bacterial cluster over time, and we speculate that other stimuli like bacterial products may contribute to the increase in *Socs3* expression (36). We have previously shown that *Socs3* expression induced by Mtb in macrophages is dependent on NF-κB and thereby at least partially *Il-6* and *Il-10* independent (21). It is worth mentioning that the local expression of immune transcripts in the bacterial cluster (**Figure 5**), as measured here by ISS, can differ from the bulk measurement of cytokines, e.g., in lung homogenates. Therefore, comparison of ISS data with a measurement of cytokine levels in the lungs will not automatically match our findings but would be an interesting follow-up.

CD11c is commonly used to identify DCs but can also indicate the presence of alveolar macrophages, monocytes, and immature macrophages. DCs can take up Mtb antigens but also get infected by bacteria. Alveolar macrophages reconstitute a significant proportion of macrophages within the Mtb-infected mouse lung (37). They are the first cell contact of Mtb with the host after inhalation and contribute to Mtb dissemination, which matches our finding of an enriched *Cd11c* expression at subcellular distances at 3 wpi but not later (1). At 12 wpi, a transcript pattern indicating activated and antigen-presenting cells (*Cd80*, *Cd86*, and *Il12p40*) was preferentially found in uninfected cells. This is in line with previous observations showing a negative spatial association of CD11c+ cells with Mtb bacteria in mouse lungs in contrast to a positive correlation of CD11b+ cells indicating macrophages in neighborhood analysis (14, 38). It remains to be investigated whether these non-infected CD11c+ cells contribute to bacterial control. Interestingly, elevated levels of *Cd11c* and transcripts indicating dendritic cell activation (*Cd86* and *Il12p40*) were found in SC in contrast to BOG of C3HeB/FeJ mice confirming their functional diversity.

### Mtb and Adaptive Immunity

It has been long questioned if a granuloma structure enables the direct contact of primed T cells with infected macrophages as required for their activation. Recent studies redirected the focus of T-cell functionality in, e.g., lung homogenates to the importance of the localization of T cells close to Mtb-infected cells to execute their function (16, 17, 39). In the single bacteria pipeline, we expected a preferential accumulation of T-cell transcripts especially indicating a differentiation of macrophage activating CD4 Th1 cells (e.g., *Cxcr3*, *Ifng*, and *Tbx1*) at a 10- to 30-µm distance compared to further distances. This would have indicated a functional immune interaction between an antigen-specific T cell and an Mtb-infected macrophage. However, we did not confirm this hypothesis. Instead, for 8 and 12 wpi, an increase in T-cell transcript frequencies for 10–30 µm and 30–100 µm correlated with the onset of adaptive immunity without a localized accumulation. Contradicting our expectations was the reduced T-cell transcript density at 12 wpi vs. 3 wpi within the bacteria cluster. The BOG formed in C3HeB/FeJ mice resembles human necrotic granulomas despite their susceptible genetic phenotype and will be discussed in detail below.

### C3HeB/FeJ and C57BL/6 Mice

C3HeB/FeJ mice are very susceptible to Mtb infection despite having a functional immune system with unimpaired CD4 Th1 cell differentiation and macrophages that respond to IFN-γ with NO production (40). Their survival is shorter than iNOS-deficient mice and susceptibility has been mapped to the *sst1* locus and the interferon-inducible gene *Ipr1* that switches the mechanism of cell death in macrophages (41, 42). C3HeB/FeJ mice are used as a TB infection model as they develop necrotic granulomas not seen in C57BL/6 mice, and this severe pathology within a generally immunocompetent mouse resembles in that aspect human TB (20). The formation of necrotic granulomas in C3HeB/FeJ mice is not a consequence of high bacteria loads, as it has been shown to precede the strong increase in bacteria amplification. The comparison of bacteria containing parts of necrotic granulomas (BOG) that were identified by the bacteria cluster pipeline to the non-necrotic small bacteria cluster (SC) showed a different immune profile within the cluster types. It also confirmed the ability of macrophages to induce *Inos* and *Tnf.* Our analysis clearly demonstrates the compartmentalization of immune parameters looking at SC and BOG, respectively (**Supplementary Figure 6**). However, it is important to bear in mind that the mechanisms leading to necrotic granulomas without control of bacteria growth in C3HeB/FeJ mice may differ from local immune events leading to the progression from latent to active TB in humans. BOG clusters were characterized by an increased expression of *Foxp3*, *Cd8a*, and *Ifng* transcripts previously identified as characteristic for necrotic centers in these mice (13). It is uncertain if *Cd8a* indicates the presence of T cells as *Tcrb* and *Cd8b* levels are low and *Cd8a* may also be expressed by dendritic cell subsets in mice during infections (43). As previously indicated, the low signal density did not allow a cell phenotyping with several markers detected on one cell, and the absence of additional T-cell transcripts emphasized this uncertainty.

### Limitations, Future Developments, and Translation

Here, we used AR staining, which is an acid-fast fluorescent staining, a diagnostic method used as an alternative to classical Ziehl-Neelsen staining. Problems with AR stainability of bacteria in the necrotic core of C3HeB/FeJ mice at 10 wpi have been reported previously, as well as a reduction of general acid-fast staining of Mtb during dormancy in humans; a change in phenotype/cell wall composition was suggested as a reason for this phenomenon (44, 45). In our study, Mtb bacteria are stainable in all C57BL/6 sections and in the necrotic center at 10 wpi in C3HeB/FeJ mice, and an explanation could be that the pepsin treatment of the tissue before the ISS assay made the bacteria accessible for the staining. As a long-term goal, we would like to combine the detection of immune transcripts with mycobacteria transcripts in our ISS assay, to detect transcripts indicating viability, stress responses, and replication of Mtb in connection to their immune environment. The technical challenge in that approach is to digest the mycobacteria cell wall to make mycobacterial RNA accessible for detection probes without damaging the surrounding tissue and degrading host RNA.

Our study is based on a padlock probe panel against 33 immune transcripts of selected immune cell markers, activation molecules, and cytokines, which were all identified in parallel within one section. Although cell typing would likely reveal even more details of the immune environments, our focus was to study the expression levels of immune transcripts, in relation to the Mtb bacteria localization in the infected mouse lung tissue and not doing cell typing per se. Cell typing would require a higher density of transcript signals as achieved in our samples in combination with a much larger padlock probe immune panel, in order to base cell identity in multiple marker reads per cell. The nature of our samples, which are formalin-fixed and paraffin-embedded (FFPE), a biosafety requirement for the collection of Mtb-infected tissues, shows lower signal densities compared to fresh frozen samples. Methodological advancements have been achieved since the beginning of our study, e.g., the direct RNA detection for ISS (46), that would allow for increased transcript detection and subsequent cell typing in the future. However, we want to emphasize that ISS has been shown to be highly reproducible and representative for transcripts even at low signal densities (13, 26, 47) and that several transcript probes have been confirmed by immunohistochemistry in our previous publication (13).

We demonstrate here the advancement of combining ISS data (identified transcripts as coordinates) and bacteria localization. ISS was conducted first and the same slide was used for bacteria (AR) and HE staining. The fact that all the experimental procedures are conducted on the same slide allows us to acquire the exact coordinates of transcripts or bacteria localization and connect them with the tissue histopathology. However, if coordinates for identified cells by immunohistochemistry are available, these can be used as input in the pipelines as well. High multiplexed immunohistochemistry could not be performed on the same tissues in our case, since the processing with multiple experimental techniques (ISS, AR, and HE staining) could have possibly affected the structure of several antibody epitopes.

Altogether, the here-presented tools constitute a toolbox for analysis of biologically complex tissues and have the potential to increase our understanding of the localization of immune components with respect to Mtb. The advantage of using formaldehyde-fixed paraffin-embedded tissues allows the use of archived human biopsies. As padlock probes are custom-designed based on target sequences, this analysis is open to all species with known target sequences.

Aiming for the translational goal to not only understand the local immune control of Mtb in mice but also predict disease progression and status in human TB patients by investigating the peripheral blood, we suggest the following next steps. Using the here-presented approach, Mtb immune environments correlated to Mtb control or progression could be defined by studying non-human primate (NHP) samples that show a spectrum of latent and active granulomas and stainable Mtb bacteria. NHPs mirror the morphology and physiology observed in human TB disease, and immune correlates of progression in lungs of NHP could be correlated to immune correlates in the blood of TB progressors (48, 49). In addition, studies comparing BCG-vaccinated and non-vaccinated animal groups would allow the definition of differences in the Mtb environment induced by vaccination and correlated to bacterial control. Examples of human granuloma samples could be applied to confirm findings of identified expression patterns in Mtb immune environments in the different conditions. *In vitro* generated granulomas would give the opportunity to manipulate and challenge the effect of identified protective expression patterns. Although important progress in the *in vitro* granuloma field has been achieved, generating, e.g., three-dimensional spheroid human granulomas forming single organized structures consisting of human lung-derived alveolar macrophages surrounded by layer of autologous T cells to study innate and adaptive stages of the TB granuloma, the reduced complexity of *in vivo* granulomas must be considered (50, 51). Lastly, a transcriptomic meta-analysis of peripheral blood from patients with active and latent TB could correlate identified patterns of Mtb’s immune environments during disease progression to systemic measurable parameters.

Overall, our here-presented tools allowed for spatial analysis of transcript expression according to the tissue histopathology and the causative agent (Mtb) localization, which enabled us to compartmentalize the disease lesions and their distinct immune environments. As the identification of bacteria cluster is based on fluorescence intensity, the analysis pipeline is not limited to Mtb infections. It could be applied in other infectious diseases in which pathogens can be visualized in tissues either by direct staining or by antibody- or hybridization-based detection, enhancing our understanding of tissue and disease heterogeneity. Another application could be in the cancer field, in which a combination of transcripts identified by ISS with areas overexpressing tumor antigens like c-myc and p53 detected by immunohistochemistry would lead to a better definition and understanding of tumor microenvironments.

## Data Availability Statement

The raw data supporting the conclusions of this article will be made available by the authors, without undue reservation.

## Ethics Statement

The study was performed under the approval of the Stockholm North Ethical Committee on Animal Experiments (permit number N397/13 and N487/11).

## Author Contributions

BC conceived the study. AM and BC developed the methodology of the study. CY and BC performed experiments. AM, TPS, and BC analyzed the data. AM, XQ, TPS, SMS, and BC developed software. CY, MN, MER, and BC contributed reagents and materials. MN, MER, and BC acquired funding. AM and BC prepared the figures and wrote the paper. MN and MER critically revised the article for important intellectual content. All authors contributed to the article and approved the submitted version.

## Funding

This study was supported by the Karolinska Institutet (BC and MER), and the Swedish research council (grant 2019-01238, https://www.vr.se/) and Cancerfonden (grant CAN 2021/1726, https://www.cancerfonden.se) to MN. The funders had no role in study design, data collection and analysis, decision to publish, or preparation of the manuscript.

## Conflict of Interest

MN is an advisor for the company 10x Genomics.

The remaining authors declare that the research was conducted in the absence of any commercial or financial relationships that could be construed as a potential conflict of interest.

## Publisher’s Note

All claims expressed in this article are solely those of the authors and do not necessarily represent those of their affiliated organizations, or those of the publisher, the editors and the reviewers. Any product that may be evaluated in this article, or claim that may be made by its manufacturer, is not guaranteed or endorsed by the publisher.
