# Supplementary material for: Spatial Resolution of Mycobacterium tuberculosis Bacteria and Their Surrounding Immune Environments Based on Selected Key Transcripts in Mouse Lungs
Source: Front Immunol. 2022 May 18;13:876321. doi: 10.3389/fimmu.2022.876321 (PMC9157500; doi:10.3389/fimmu.2022.876321)
Supplement: Supplementary file 1 [file DataSheet_1.zip › Supplementary material_Rev/Legends-Supplementary Figures_Rev.docx]

### Legends-Supplementary Figures

**Immune environments of *Mycobacterium tuberculosis* bacteria**

*Supplementary Figure 1*

1. Hematoxylin-Eosin (H/E) and DAPI stain of one ISS-analyzed lung tissue section of C57BL/6 mice at the 12 weeks post Mtb infection (wpi) is shown. ISS signals for indicated transcripts were plotted on Auramine-Rhodamine-T (AR) stained image as background. Threshold: 0.35, Scale bar: 1000 μm
2. Comparative plotting of *Cc10*, *Inos* and *Cd19* transcripts at 0.35 and 0.45 thresholds (TH) for base identification during barcode sequencing. ISS signals are plotted on a DAPI background of one C57BL/6 lung section taken at 3 wpi. Scale bar: 1000 μm
3. Linear regression of transcript reads at TH 0.35 and 0.45 shows a large positive correlation (r^2^=0.97).
4. Frequencies of indicated transcripts among the sum of all immune transcripts in three sections of C57BL/6 mice at 3, 8 and 12 wpi and of C3HeB/FeJ mice at 10 wpi were calculated. Log2 mean values ± SEM are displayed, differences that were significant to 3 wpi are indicated (* for p<0.05, Student’s t-test).

*Supplementary Figure 2*

1. Normalized confusion matrix after machine learning (fast gentle boosting) of cell profiler analyst shows the frequency of true prediction in the three classes: single bacteria, bacterial clumps and background.
2. Comparison of frequencies of indicated immune transcripts at 12 wpi in different distances of single bacteria and bacterial clumps.

*Supplementary Figure 3*

Exemplary input and output of bacteria cluster pipeline for one C57BL/6 section at 3 wpi (A) and 8 wpi (B) shows the AR image (100%, left panel), cluster identification on the 20% subtracted image (middle) and applied 100 μm gradient around identified clusters (right). Scale bar: 1000 μm

*Supplementary Figure 4*

Transcript densities at indicated distances to bacteria were identified in three sections per condition and normalized to the respective transcript frequency of the total section at (A) 3 and 12 wpi in C57BL/6 mice and (B) BOG (Big, Organized Granulomas), IOG (Intermediate, Organized Granulomas) and SC (Small clusters) of 10 wpi C3HeB/FeJ mice Selected transcript densities are displayed: (A) *Inos, Cd68, Tnf, Socs3, Il6, Il10, Tcrb, Ifng, Cd8a, Cd8b1, Cd4* and *Cxcr3* and (B) *Inos, Cd68, Cd8a, Cd8b1, Tcrb, Foxp3,* *Socs3* and *Ifng*. The mean of log2 ratio ± SEM is displayed, and significant differences are indicated (ANOVA, * for p<0.05, ** for p<0.01, ***for p<0.001 and **** for p<0.0001).

*Supplementary Figure 5*

1. Additional two regions showing annotations of cluster types based on H/E images in C3HeB/FeJ sections at 10 wpi (left panel). Classification into BOG: big organized granulomas, IOG: intermediate organized granulomas (less pronounced encapsulated structure) and SC: small cluster. Cluster identification based on AR image is displayed (middle) and applied 100 μm gradient around identified clusters (right). Scale bar: 1000 μm
2. Heatmap analysis of transcript density ratios of annotated BOG (n=4) and SC (n=9) is shown.

*Supplementary Figure 6*

Graphical representation of the experimental and analytical workflow and main findings for the bacteria cluster analysis in Mtb-infected C57BL/6 and C3HeB/FeJ mouse lungs. At 10 wpi C3HeB/FeJ lungs, distinct cluster types were identified: BOG (big, organized granulomas), IOG (intermediate, organized granulomas) and SC (small clusters) and only the latter was identified at 12 wpi C57BL/6 lungs. The main differences in transcript densities are highlighted for SC and BOG. The transcript expression of IOG showed an overlap between SC and BOG and is not shown. Scale bar: 1000 μm
