# Supplementary figures and images for: Spatial Resolution of Mycobacterium tuberculosis Bacteria and Their Surrounding Immune Environments Based on Selected Key Transcripts in Mouse Lungs
Source: Front Immunol. 2022 May 18;13:876321. doi: 10.3389/fimmu.2022.876321 (PMC9157500; doi:10.3389/fimmu.2022.876321)

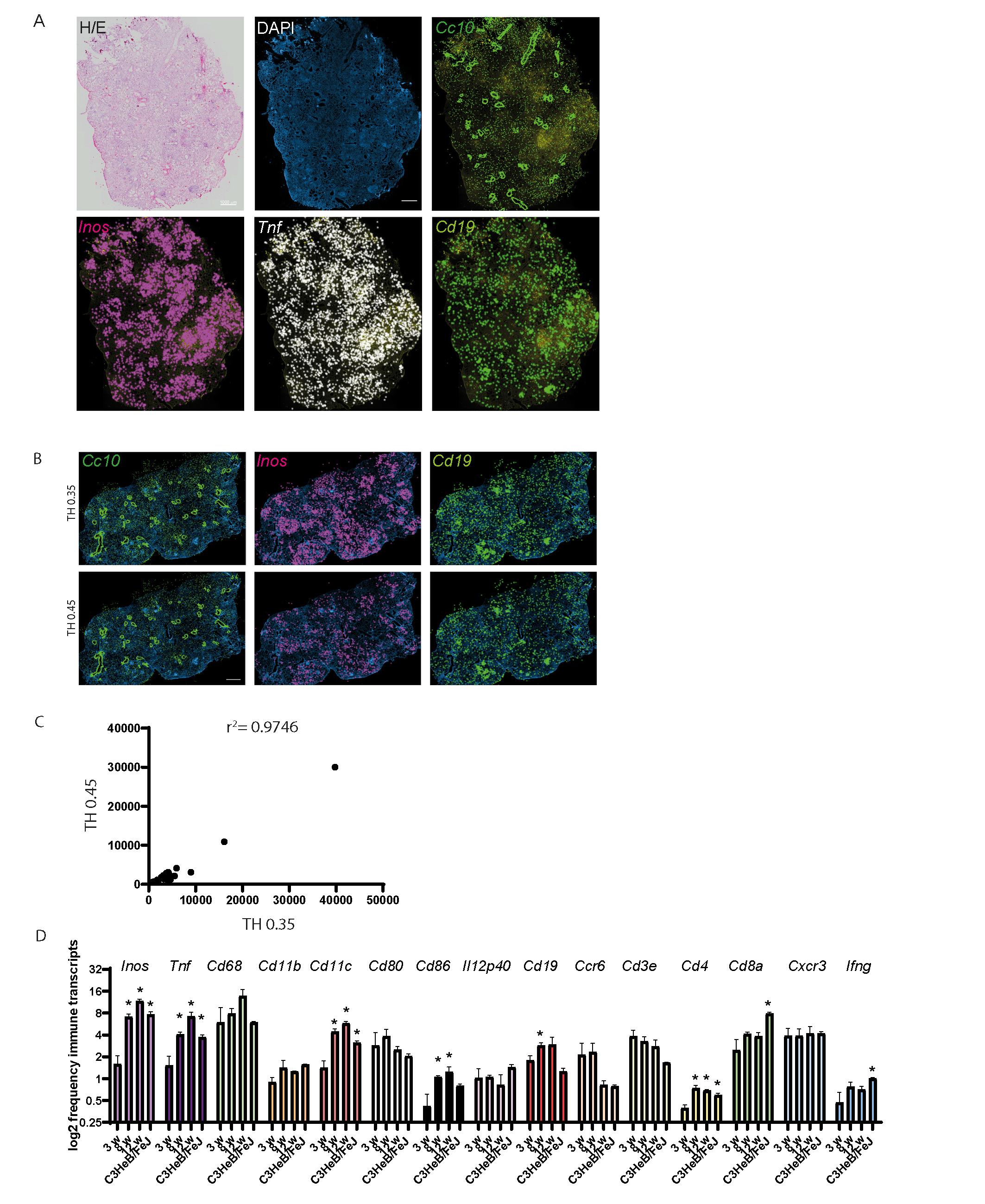

Supplement: Supplementary file 1 [file DataSheet_1.zip › Supplementary material_Rev/Sup Fig1.tif]

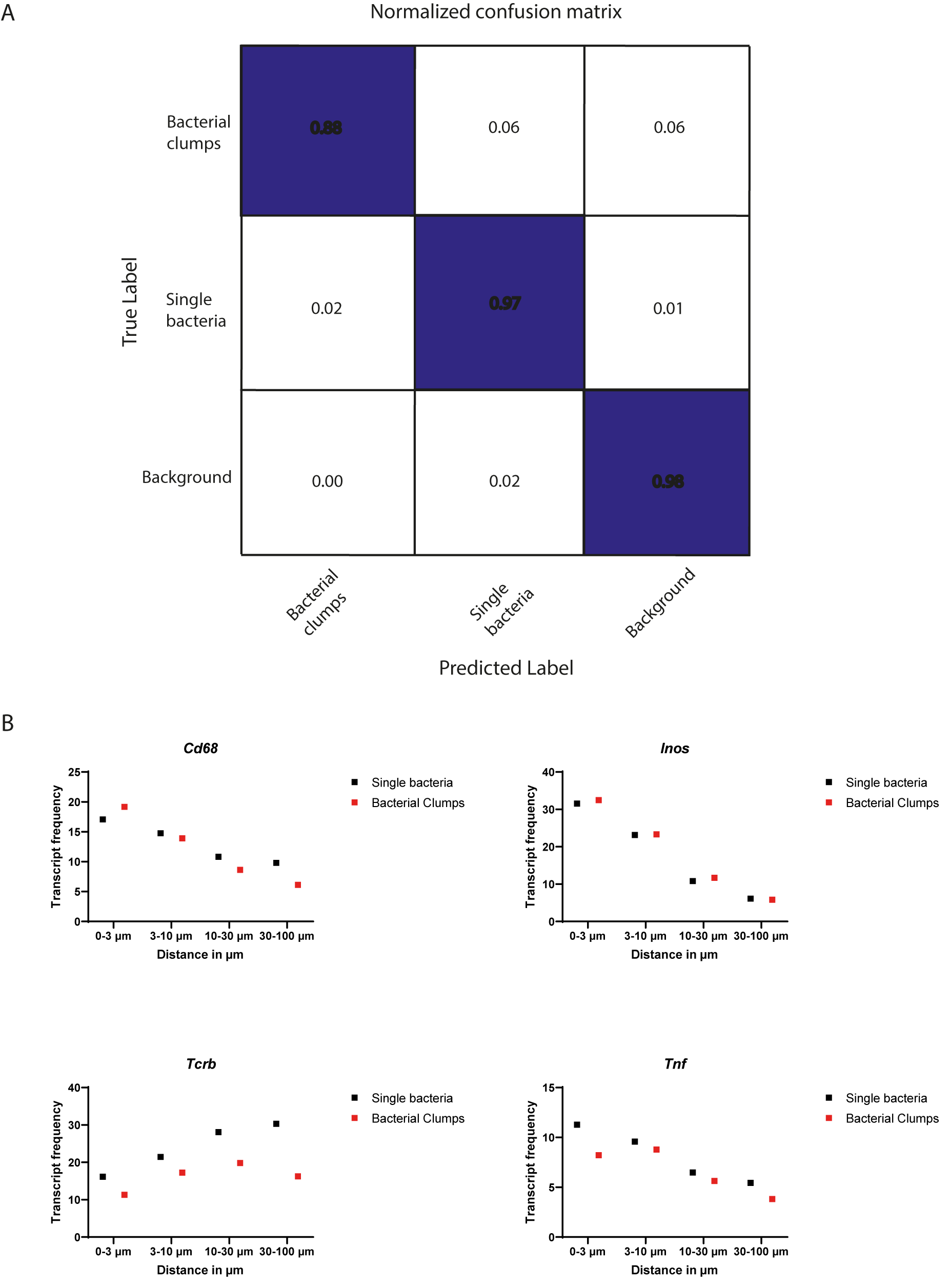

Supplement: Supplementary file 1 [file DataSheet_1.zip › Supplementary material_Rev/Sup Fig2.tif]

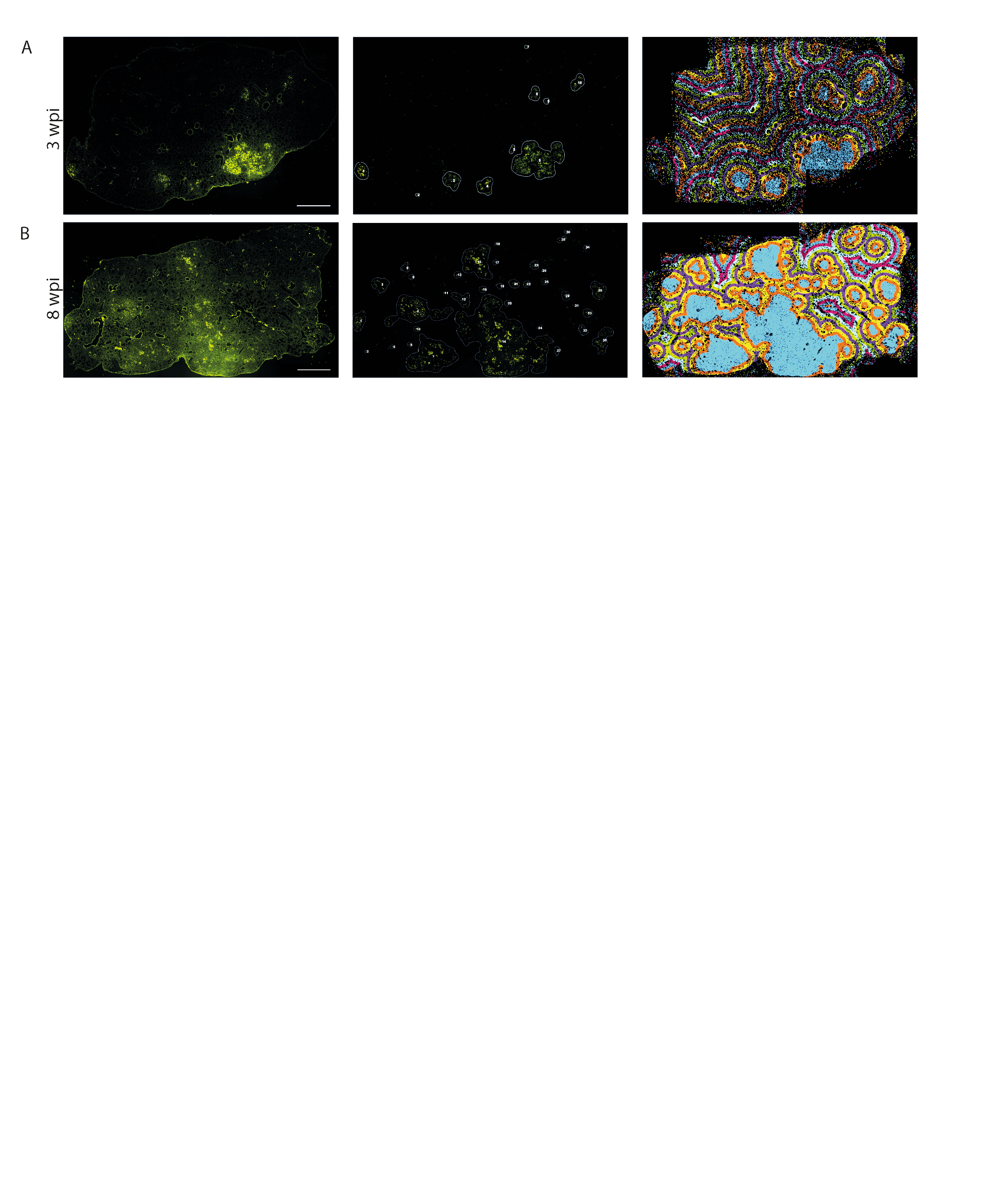

Supplement: Supplementary file 1 [file DataSheet_1.zip › Supplementary material_Rev/Sup Fig3.tif]

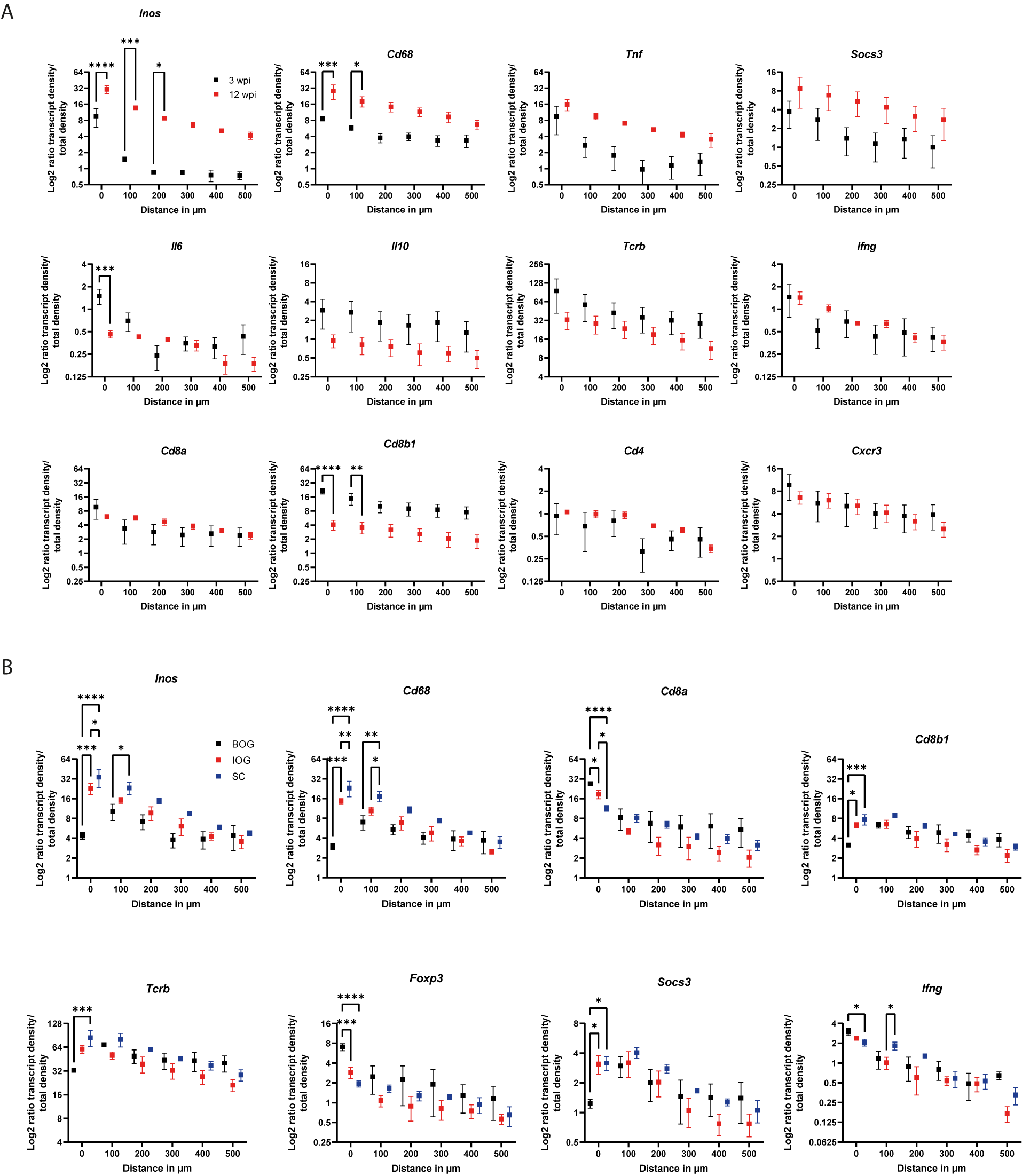

Supplement: Supplementary file 1 [file DataSheet_1.zip › Supplementary material_Rev/Sup Fig4.tif]

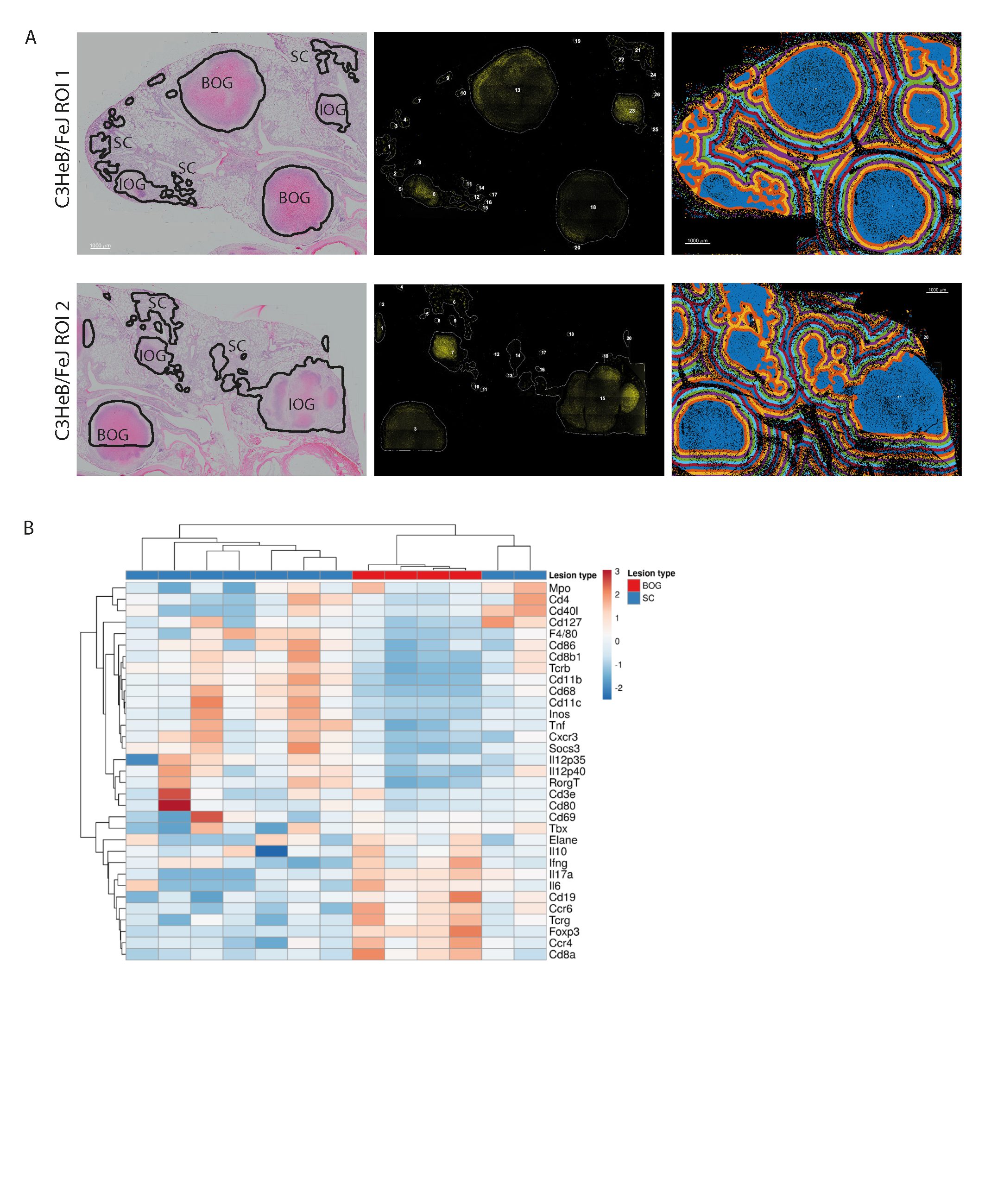

Supplement: Supplementary file 1 [file DataSheet_1.zip › Supplementary material_Rev/Sup Fig5.tif]

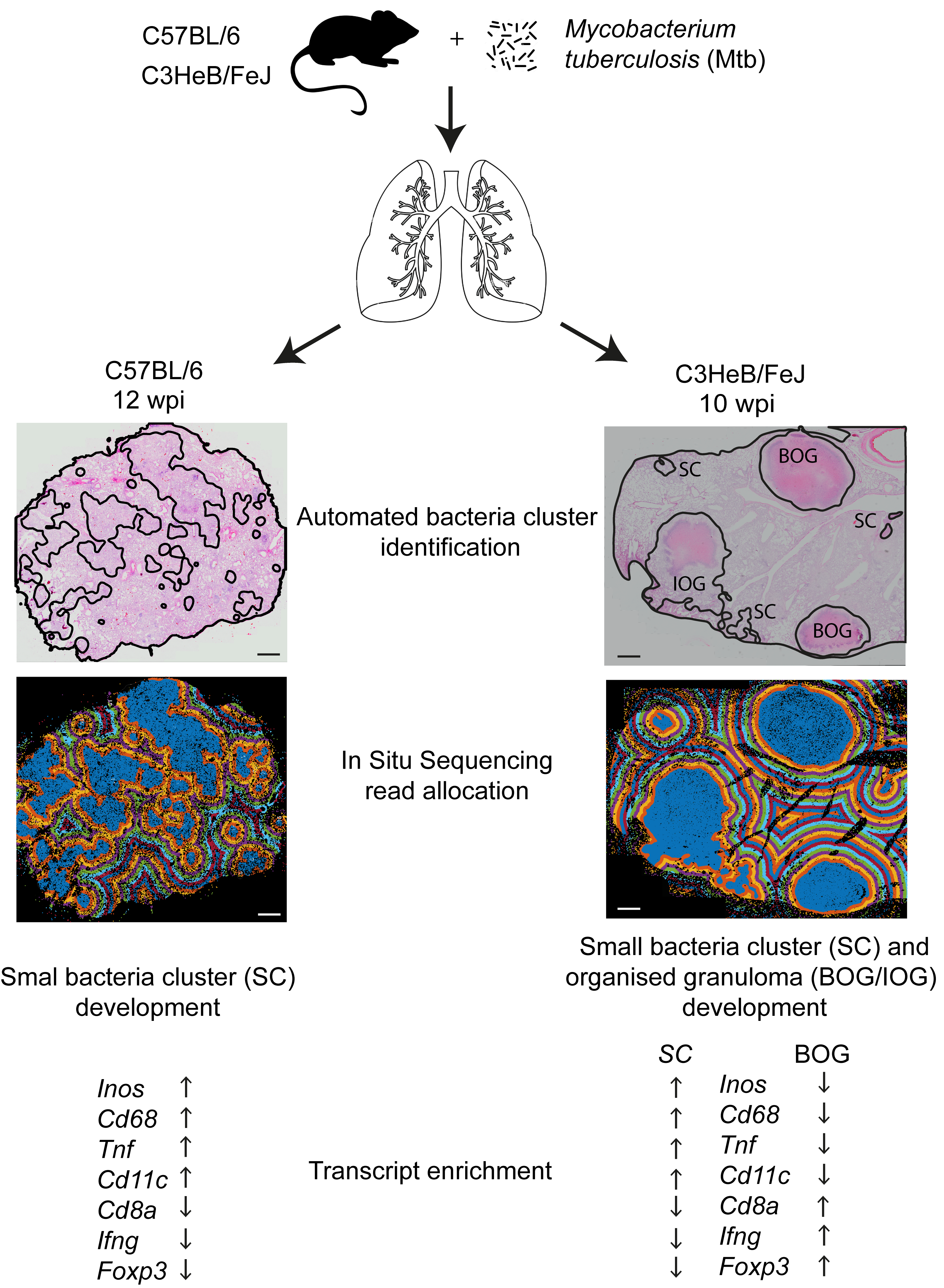

Supplement: Supplementary file 1 [file DataSheet_1.zip › Supplementary material_Rev/Sup Fig6.tif]
